# Fog computing at industrial level, architecture, latency, energy, and security: A review

**DOI:** 10.1016/j.heliyon.2020.e03706

**Published:** 2020-04-08

**Authors:** Gustavo Caiza, Morelva Saeteros, William Oñate, Marcelo V. Garcia

**Affiliations:** a Electronic Engineering, Universidad Politecnica Salesiana (UPS), 170146, Quito, Ecuador; b Department of Systems Engineering and Automation, Basque Country University (UPV/EHU), 48013, Bilbao, Spain

**Keywords:** Computer science, Industry 4.0, Cloud computing, Fog nodes, Fog computing, Smart factories

## Abstract

The industrial applications in the cloud do not meet the requirements of low latency and reliability since variables must be continuously monitored. For this reason, industrial internet of things (IIoT) is a challenge for the current infrastructure because it generates a large amount of data making cloud computing reach the edge and become fog computing (FC). FC can be considered as a new component of Industry 4.0, which aims to solve the problem of big data, reduce energy consumption in industrial sensor networks, improve the security, processing and storage real-time data. It is a promising growing paradigm that offers new opportunities and challenges, beside the ones inherited from cloud computing, which requires a new heterogeneous architecture to improve the network capacity for delivering edge services, that is, providing computing resources closer to the end user. The purpose of this research is to show a systematic review of the most recent studies about the architecture, security, latency, and energy consumption that FC presents at industrial level and thus provide an overview of the current characteristics and challenges of this new technology.

## 1 Introduction

The fast development of technology has contributed to the increase of devices connected to the cloud, which generates large amounts of data. The cloud is the biggest data unit where processing and storage is performed with the main goal of making use of services and resources sought by the customers [1]. In 2015, the number of connected devices was 15.41 billion, later in 2017 it was of 20.35 billion, and it is expected to be 30.73 billion in 2020 [2]. These services and applications are used in different scenarios in the world such as Smart Factory, Smart Farming and Smart Cities [3]. Due to this rapid increase of data, a large amount of storage is required, which has generated a greater bandwidth consumption and high latency in data processing.

The development of portable computing processes, more intelligent measurements in homes/cities and vehicles, and wider wireless sensor networks has made everything interconnected through the IoT [4]. Due to the number of interconnected devices, technology is generating big quantity of data that are processed, filtered and analyzed in the cloud causing problems related with traffic congestion, delays, and privacy concerns [2], [5]. Many limitations also emerge since the cloud is unable to support some of the needful such as heterogeneous devices, low latency, mobility, and location recognition [6]. In addition, the current infrastructure is not designed for the quantity and speed of data generated by IoT [7], which has generated a high consumption of resources and a decrease in the quality of services for the end user. Besides, industrial applications in the cloud do not meet requirements of low latency, network traffic reduction, and reliability [8] which are parameters established for industrial processes where the process variables must be continuously monitored. The fog computing can be contemplated as a new component of the smart factories. [9].

The next industrial revolution is based on the combination of several innovative technologies [10], with which factories are becoming increasingly intelligent and efficient [11], providing availability and scalability to the processes by having technology in the cloud. In this way, IIoT allows connecting not just the internal manufacturing chain, but also other factories for a more vertical and horizontal integration [12].

In relation to the above, FC is being devised for facing the problems caused by IIoT, and to offer new applications and services that provide distributed solution for real-time data processing [13], as an advanced technique to reduce latency and congestion in the benefit of IIoT networks [14]. In addition, this technology enhances cloud computing by providing small platforms situated at the edges to operate the services of control, monitoring, communication, processing, settings, storage, measurement, and administration [6]. The nodes reduce the cloud load and improve processing by evenly distributed functions according to availability [15]. When a device generates data, not all data must be transmitted to the cloud, so, a part of the processing will be moved to the edge devices to reduce the amount of information to be sent.

In this way, FC has arised to address the need for real time, location recognition, geographic distribution, robust mobility, wireless access, real-time transmission, and heterogeneity [16]. At the same time, it presents new challenges in addition to those inherited from cloud computing at industrial level where current systems are already connected with proprietary software and hardware, and the interface with these inherited designs is a key blocking factor that must be considered.

This article shows a systematic review of FC regarding the architecture, security, latency, and energy consumption at industrial level, highlighting the techniques used for each of the mentioned parameters and showing the advantages and challenges they present through a review of the more recent studies in relation to the topic.

The article is organized as follows: Section 2 describes the methodology; Section 3 shows the key concepts of this research work; Section 4 presents the studies developed in theses topics; Section 5 presents the discussion and finally, Section 6 presents the conclusions and future studies.

## 2 Methodology

The research was conducted by selecting the most recent works since 2016 to 2019, in high-quality bibliographic databases such as Science Direct, Institute of Electrical and Electronics Engineers (IEEE), Taylor & Francis, SpringerLink, Google Scholar, and Scopus database.

Identifying articles for the first group started with extensive inquiries about databases of scholarly literature of a series of topically essential keywords. For instance, the following keywords were used, typically in sets of two and groups of three or more as well: [“cloud computing”], [“fog computing”], [“big data”], [“smart manufacturing”], [manufacturing], [“IIoT”], [“industry 4.0”], and [“WSN”].

Studies implemented under different protocols for IoT applications and subsequently exploring IIoT applications were chosen. The most relevant problems for the development of FC were established, and it was determined that studies of other authors focus on 4 parameters that are: architecture, latency, security, and energy consumption. These parameters are the ones that must be improved since they are critical for the process operation at industrial level due to the variables and quantity of data they handle.

However, the first part of the paper shows a quick review of the characteristics and concepts that must be considered for the development of FC in industrial scenarios. Subsequently, the applications and characteristics implemented in cases of non-critical studies where architecture, latency, security, and energy consumption are not a problem, are taken as a basis to later analyze the possibility of implementing it at industrial level using techniques or protocols that have been used before, demonstrating good functioning in IoT and the accomplishment with the FC parameters.

The second part presents the studies that focused the IIoT applications and the treatment given to each problem in the parameters. The aim of this research is to present a systematic review of the most current studies analyzing the architecture, security, latency, and energy consumption of FC at industrial level and thus provide an overview of its current characteristics and challenges.

## 3 Overview of key background concepts

In this section, we briefly give an overview of the key concepts to understand the development and characteristics of fog computing to industrial level

### 3.1 Cloud computing

Cloud platforms present the prerequisites of especially data storage for big data approaches since they provide scalable and computing power. [17]. Cloud computing has been considered as a key enabler to meet the requirements of IIoT applications [18].

### 3.2 Fog computing (FC)

FC was defined by Cisco as an extension of cloud that goes from the center to the edge to enhance frequent services, low latency and big data analysis [19], [20]. It consists of a big number of fog nodes (FNs) geographically implemented in different places to provide data services and applications [21], where each server is a lightweight version of the cloud server and thus provides resources closer to the devices [22], [23]. It allows them to cooperate and communicate with each other, forming a network to execute storage and processing functions in real time [24], [25].

In addition, the hardware and software of the fog nodes can be designed according to the requirements of an application and the industry-specific security and communication standards [26], thus FC provides local processing support with acceptable latency for a company and, since the data are not structured, the fog can refine them locally, before sending them [27]. Due to these characteristics, FC performs applications locally, providing a scalable data storage for improving the processing and support capacity.

FC is not intended to take the place of cloud computing but to complement it, thereby reducing the burden on the cloud [5]. It supports IoT applications, presents low latency, mobility, location recognition, scalability, security, and integration with heterogeneous devices [15]. It also avoids traffic between the cloud and users for saving network bandwidth, reducing energy consumption, and using resources according to the needs [25].

The nodes offer computing, storage, and networking resources for the applications that operate under this infrastructure [28]. These are heterogeneous devices that range from servers, access points, base stations, edge routers to final devices such as: smart sensors, actuators, etc [19]. The nodes can be internally scaled by adding hardware or software [29], or externally, adding as many nodes as needed for providing a localized service, thus distributing cloud service tasks at each node and improving scalability and redundancy. Performance is affected by the location and allocation of resources given to each node [30].

### 3.3 Internet of things (IoT)

IoT is a set of networks in which each device is connected, which involves managing a large amount of information that requires to be stored and efficiently processed, and must be delivered to users in a safe and understandable. A solution for the storage and processing of information is to adopt cloud computing technology [31]. This technology provides computer resources such as high-speed internet connection, efficient data storage, computing power [32] where users can access information anywhere in the world, so the cloud computing is the basis for Internet of Things [33]. IoT devices do not center on the reliability or connectivity that is necessary for an industrial environment [34], since they are too relatively “vulnerable” to hackers due to resource limitations [35].

### 3.4 Industry 4.0

**Figure 1:**
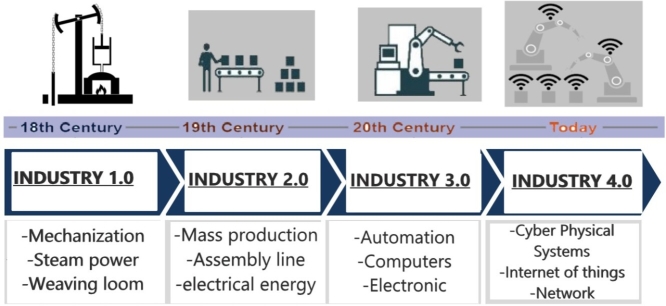
History of Industrial Revolution [42].

Industry 4.0 in Europe or Advanced Manufacturing in North America [36] refers to the fourth-generation industry that focuses on the manufacturing industry [27], enabling suppliers and manufacturers to take advantage of the new technological concepts to obtain new or improved products/services with massive customization, lower costs, and higher productivity, this due to the increase in innovation cycles, and autonomous data exchange between systems [37]. It also provides several possibilities within the transformation in the industry. While currently, one fifth of industrial companies have digitalized their processes, in approximately five years 85 percent of companies will have implemented industry 4.0 solutions [38], that is why the digitalization of the industry is considered the present and the future. Companies generate a large amount of data from the manufacturing and production process, which requires real-time compilation and analysis [39], [40], so cybernetic industrial systems are the main enabling technology for advanced manufacturing, which refers to the creation of manufacturing intelligence through the use of generalized networks in real time and operational data flows [41]. (See Fig. 1.)

### 3.5 Wireless sensor networks (WSN)

The wireless sensor network is highly used to obtain information from a specific environment, however, it presents several challenges among which are: applications, single network topology, traffic characteristics, and severe limitations of energy resources [43] due to the topologies and remote locations of the sensors, which are powered by batteries and their useful life depends on them. In addition, these networks are widely used in intelligent transport, the monitoring of medical care and, especially, in the industrial field, where they are called Industrial WSNs (IWSNs) [44], which require a trustworthy service in data processing and information exchange [45].

### 3.6 Industrial Internet of things (IIoT)

In present years, IoT has been used in the industrial field and has shown that it does not meet the requirements for applications such as: latency, reliability, and security of data [8], therefore Industrial IoT appears [46]. IIoT implements an integrated technology to collect information and transmit it to data centers, updating the parameters in the form of a closed-loop system [47]. It is also known as the future of the strategic large companies [48] to build applications that are characterized by a high degree of integration and flexibility, allowing to optimize the production line with better quality, energy efficiency, prediction of failures, product planning, and prediction of resources [26]. IIoT may involve interactions with cyber physical system (CPS), robots, sensors, controllers, actuators, radio frequency identication (RFID), global positioning systems (GPS), cameras, etc. [49] with an architecture that is evolving from the centralized to the distributed cloud [50]. The intelligent manufacturing requires real-time data processing so, reliable communications are a key factor since the congestion or delay in the information can cause interruptions of the process. In this way, the industrial environment is facing several challenges because individual solutions for both hardware and software, and the reuse of applications is not possible due to the lack of distribution platforms [51].

IIoT collects data at an industrial level through various devices for information detection [43], that is to say, about more computing resources, networking, storage, and intelligence for IIoT devices, and provides various benefits such as: rapid response, big data management, traffic reduction, and ultimate intelligence [23]. In the current IIoT data processing system, there are two main forms of computation: Distributed computing which indicates that each sensor processes each data, and centralized computing where the information is transmitted to the data center that performs the processing [52], [53]. It should be noted that the amount of industrial connected elements increases the IIoT services, which is the reason why more and more computing resources and communication are requested, which leads to a bottleneck in terms of data latency and traffic overload [54].

## 4 Conducted studies

In this section, we focus on analyzing the work done by other authors to obtain the characteristics and techniques used to solve the problems that fog computing presents. The industrial implementation of FC is becoming an interesting and promising research axis for factories, the characteristics and challenges existing at the industrial level have been reviewed where the parameters analyzed were architecture, latency, security and energy consumption because these are critical conditions in the industry.

### 4.1 Architecture

**Figure 2:**
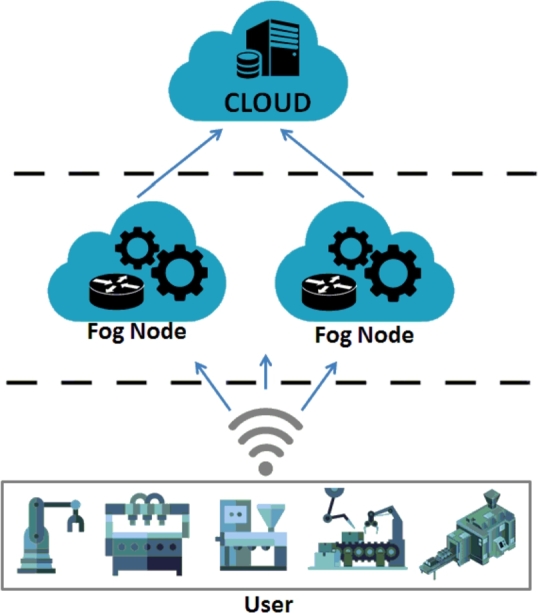
General Architecture of Fog Computing [55].

A fog computing architecture is usually divided into 3 layers [9], [56]. Fig. 2 illustrates the fog computing topology, the cloud platform stores the information of the production for different engineering applications, which are published and executed by fog nodes deployed within the facilities' local network. These local nodes offer data security and privacy, these communications depend on the facilities' existing security policies and services [41].

In [57], authors present an application for remote detection, monitoring, and scalable computing for real-time diagnosis using WSN, cloud computing, and automatic learning, obtaining experimental results to monitor vibrations and energy consumption even though they do not take advantage of the potential of a FC architecture. In [3] the authors analyzed in three main edge computing reference architectures aimed at industry 4.0 and based on the ISO/IEC/IEEE 2010: 2011 standard presented by some most influential technological working groups. These architectures center on using edge computing as a solution for the capabilities of your cloud-based technology implementations for big data. The authors have established a great difference in the development of architectures related with other scenarios that are not related to industry 4.0. This architecture will have as main aims to attain in less time data analysis in the local device.

In [58], the authors have developed a wavelet construction method using Embedded C where the IIoT devices with limited resources can successfully participate in distributed computing at the edges of the IIoT network. The Morlet wavelet is built for the C28x real time digital signal processor (DSP). In the Fog the wavelet is mixed with a sanded signal and network signals electric. They use matlab to reduce the signal noise, identify and examined signals, the result of the convolution shows that the noise in the examined signal is reduced using this method. In [59], the authors propose a CPS system analysis based on graphics and parameters that allows more security, reliability and categorization of fog computing. This system is based analysis and development to improve the latency, security and other QoS parameters, as well as to allow for better interoperability and optimal use of technology in the industry. Experimental analysis shows that the systems have a variable set of parameters and architectures, while a composite system can mimic conducts of random network models.

In [24], the authors propose the implementation of an intelligent computer system consisting of a center in the cloud, gateways, fog devices, edge devices, and sensors, and the deployment of the system using a bioinspired approach based on a monkey algorithm and genetic algorithm that minimizes the total cost of the installation. Results showed that the system performs well in logistics centers of moderate size. In [53], the authors determine the challenges facing the machine-to-machine communication (M2M) in industrial systems and present a M2M mechanism oriented to data based on zeroMQ to deal with the problems of heterogeneity and ubiquitous access to data in industrial applications. The results show the feasibility of the suggested mechanism due to its flexibility to deal with the hierarchical architecture and heterogeneity. In [60], a survey on IoT on a large scale petrochemical plants was presented, as well as the recent activities in standards of communication for the IIoT addressing middleware approaches, as an intelligent industrial ecosystem (IISE) which permit the fast deployment and integration of heterogeneous WSN with advances in crowdsensing-based services. An approach of osmotic computing, a new model to support the efficient implementation of IoT services and applications at the network edge [61], is used to solve a complex problem of deep learning and exhaustible artificial intelligence (XAI) showing that it could lead to significant savings in memory on the edge.

In [62], the authors propose a fog servers hierarchical deployment, using models of probabilistic analyses where data and IIoT applications are divided into requests for high- and low-priority also introducing a workload allocation algorithm for downloading beak loads at higher levels of the fog hierarchy. Also in [63], the authors propose a system to manage applications based on FC and compare three balancing algorithms to minimize the waste of energy and distribute the surplus along the smart grid. The referred studies take advantage of a fog-based architecture; however, they do not contemplate the low computational capacity of the end devices and the data prediction for energy savings.

### 4.2 Latency

The cloud-centric systems need devices to send their data to the cloud and wait for its response, thereby creating latency [64]. Many industrial applications cannot tolerate high latency. Researchers have thus developed some studies which have been performed to reduce latency and are shown below. In [5] the study presents a hybrid approach that combines the diffuse learning to improve service and latency. The fuzzy inference system consists in tracing past experiences and takes decisions to load the data in the cloud. In [15], an unmanned aerial vehicle (UAV) architecture is proposed based on FC for fog load balancing, where resources dynamically change depending on the availability of each location, in this way, receiving updates of the number of idle and occupied nodes, so to send the task based on the minimum latency. In [14], the authors propose a four-layers outline, which be composed of the cloud layer, the clustering layer, data programmer layer, and the device layer, showing more than 15 percent of performance gain over the optimization under different work scenarios. However, the techniques used in the studies described above have not been tested at industrial level, so these alternatives may be considered due to the real-time processing requirements that are required in industrial environments. In [65], the authors propose a smart grid and smart local grid (SLG) to obtain an available, robust and secure power supply. These support the client's real-time services using FC. In this article, a hierarchical architecture has been proposed for security purposes for data processing in both cases using fog. The authors focus on transmission delay and processing between machines, nodes and fog servers, show various techniques and optimization algorithm to reduce latency. Cloud computing is not suitable for the storage of Big Data generated by IIoT, to solve this problem, the maximum data processing functions are implemented outside the cloud.

### 4.3 Security

The information security should be considered when deploying an architecture since problems may emerge in data and networks. Public and private organizations operate in environments of cloud, osmotic, fog, and edge computing so it is important to develop a threat intelligence platform (TIP) to defend the systems against attacks [66]. At industrial level, security is the main factor since data must be protected, so, security vulnerabilities are avoided to a large extent through the use of sensors and intelligent devices. The security and privacy mechanisms that exist in cloud cannot be directly applied to FC due to its characteristics of geographical distribution, mobility, and heterogeneity [67], [68]. Among the main security problems are the authentication, confidentiality, privacy, and availability of information since these mechanisms help to establish control of access to authorized individuals or entities. In this study, we analyzed from various points the security threats and challenges that affect fog computing.

The study in [57] proposes a mechanism called fog computing intrusion detection system (FC-IDS), which is a technology that resists the distributed denial of service (DDoS). A hypergraphic grouping model is proposed, based on the A priori algorithm, for effectively describing the association between nodes that suffer DDoS threat, simulation results show that the model has better performance for resource utilization of fog nodes. The mentioned study and techniques have improved safely, but do not consider the increase in latency.

In [69], the study evaluates the performance based on both real experiments and simulations. The system monitors the temperature of a factory workshop and focuses on the data processing, safe storage using unique identity, keys on each node, efficient recovery and dynamic collection. The data are previously processed by the edge server and, then, time-sensitive data are locally used and stored, while non time sensitive data are transferred to the server in the cloud. This study is conducted to the industrial level but does not discuss if its implementation is possible in heterogeneous and decentralized systems

### 4.4 Energy consumption

In [41], the authors propose an applications in industry 4.0 to reduce energy consumption of the nodes that generally have battery restrictions in which the fog nodes are used to predict data measurements and thus reduce the performance of the devices to the control unit. They use message queue telemetry transport (MQTT) driving a FC observing that MQTT is a stable standard whose many-to-many communication characteristic makes it suitable for real-time sensor. The proposed fog architecture is a solution to the energy consumption of devices but is not tested in dynamic topologies.

When applied to IIoT, the network time protocol (NTP) presents many problems due to the energy consumption limitations, the adverse condition of the channel and the dynamic topology, need some synchronization protocols. [70]. It should be considered that NTP protocol consumes many resources and increases latency in industrial environments. In [71] the authors, review the methods used to decrease energy consumption in the WSN sensor networks; The low energy adaptive clustering hierarchy (LEACH) protocol uses some methods and techniques to solve this problem. Modern high-reach and low-consumption IoT networks achieve energy savings using limited bandwidth and intelligent modulation. In addition, the authors also address loss and lossless compression methods on the edge device, loss methods are more efficient than lossless compression methods. The referred literature is summarized in Table 1, which displays the most relevant documents used for the analysis of the architecture, latency, security, and energy consumption, providing examples of methodologies for the FC analysis at industrial level.

**Table 1:** Summary of FC at industrial level.

**Author(s)**	**Focus**
Gaolei et al. [54]	Service Popularity-Based Smart Resources Partitioning
Chekired et al. [62]	Industrial IoT data scheduling service popularity
Fu and Liu. [69]	Secure Data Storage and Searching for industrial
Kumar et al. [72]	Improving the response time using FC

## 5 Discussion

This paper presents a systematic review of FC at industrial level, analyzing the architecture, security, latency, and energy consumption. In the first part, the analysis is applied to IoT was carried out, subsequently focusing on the specific studies developed at industrial level. The first observation is that most research has focused on the development of FC for applications where latency and security are not a problem, and different methodologies have been proposed to get better the performance of the system.

Among the analyzed characteristics, the architecture highlights as it is usually divided into 3 layers. This architecture should allow the network management for distributing and enabling applications, meaning that the fog collaborates with the cloud to enable services. With the development of industry 4.0, the company must define which tasks will go to the fog or to the cloud, and allow the dynamic relocation, considering the communication interface that will determine the location of the nodes using robust and adaptive algorithms. In the same way, the services that the cloud can offer at industrial level must be established; for example: collaboration between nodes by using a load balancing algorithm to reduce the processing of low- and high-priority data, where fog computing servers also perform tasks with redundant systems. A strategy to improve the architecture would be the software defined networking (SDN) which has been developed but not implemented at industrial level.

Security is one of the main factors in the industries since they require data to be protected against attacks and avoiding problems in data and networks. It should be considered that the methods used in cloud computing cannot be directly applied to FC because of their characteristics described above.

One of the key factors that has been developed is the access control, which is being made by cryptographic protocols, authentication, confidentiality, and privacy to set access control, but these techniques consume many resources which the nodes do not feature. At industrial level, these problems are not efficiently treated, and it must be considered that most of the described techniques do not fit FC since decentralized system are more vulnerable to attacks, so new techniques must be considered. A technique that can be used is to set security on the fog nodes for access control and encryption.

The transmission and processing delays are parameters that require high attention in industrial processes since the variables must be continuously monitored. Optimization and learning algorithms, used to improve latency while maintaining the quality of service have been developed for IoT environments, however, they have not been used at industrial level with different work scenarios. It can be considered that the information must be treated previously in the cloud or on the node depending on the type of data by using virtualization techniques.

The energy consumption should be considered when implementing a FC in IIoT architecture because the nodes have battery restrictions. Protocols and algorithms have been used to reduce the performance of devices to the control unit, but the consumption of resources must be considered for increasing the latency, which has not been tested in dynamic topologies. To optimize energy consumption in the nodes, a WSN topology could be configured for the distribution according to the load, and for enabling and disabling the sensors according to the requirement

## 6 Conclusions and ongoing work

The increase of devices connected to IIoT generates a large amount of data meaning a challenge for the current infrastructure and industrial applications since they are not able to handle the data efficiently. To address these problems, a new technology called FC appears, as an extension of cloud computing for providing small platforms at the fog nodes, leading in this way with the computational resources and applications closer to the end user.

In this paper, a systematic review was performed about the most current studies analyzing the architecture, security, latency, and power consumption of FC at industrial level in order to provide an overview of its characteristics and challenges.

FC is an open field for the development of researches and applications at industrial level to improve and solve the current problems. A proposal for future studies is the development of FC under the IEC-61499 standard which would provide the characteristics of portability, interoperability, and reconfiguration of applications that are specific to the norm.

## Declarations

### Author contribution statement

All authors listed have significantly contributed to the development and the writing of this article.

### Funding statement

This research received funding from Universidad Politécnica Salesiana.

### Competing interest statement

The authors declare no conflict of interest.

### Additional information

No additional information is available for this paper.
